# A research note regarding "Variation in cancer risk among tissues can be explained by the number of stem cell divisions"

**DOI:** 10.12688/f1000research.9448.2

**Published:** 2016-11-10

**Authors:** Maxime Tarabichi, Vincent Detours

**Affiliations:** 1 IRIBHM, Université Libre de Bruxelles (ULB), Brussels, B-1070, Belgium

**Keywords:** Cancer incidence, cancer prevention, cancer etiology, cancer risk

## Abstract

Tomasetti and Vogelstein argued that 2/3 of human cancers are due to ‘bad luck’ and that “primary prevention measures [against cancer] are not likely to be very effective”. We demonstrate that their calculations for hepatocellular carcinomas overlooked a major subset of these cancers proven to be preventable through vaccination. The problem, which is not limited to hepatocellular carcinoma, arises from the general reliance of their analysis on average incidences in the United States and the omission of incidences in specific risk groups.

## Introduction

Tomasetti and Vogelstein ^1^ claimed that for tumors of relatively low incidence arising in organs undergoing many stem cell divisions “primary prevention measures are not likely to be very effective” because they arise mostly from random mutations fixed during stem cell division, independently of specific genetic or environmental factors. This conclusion—which has received much press coverage ^2– 6^—has important implications for public health and environmental research and policies. Here we re-interpret the results in light of additional data.

## Results

The authors argued that 2/3 of the variation of cancer incidence among human organs could be explained by the total number of lifetime stem cell divisions (lscd) which, according to them, drives the stochastic accumulation of random mutations. Yet, the incidence variation among organs is not informative about incidence variation among different risk groups. For example, worldwide cancer incidence variations and their association with regional risk factors are well documented ^7^. But the study by Tomasetti and Vogelstein rests mostly on current average USA incidence statistics and is therefore blind to population-specific risk factors.

**Figure 1.**
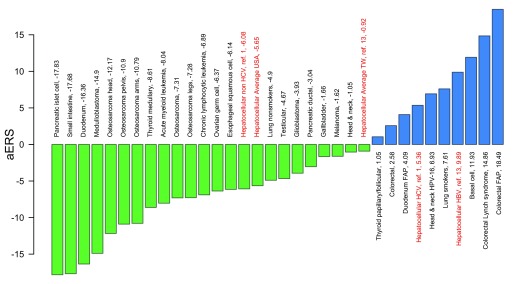
Effect of risk stratification of hepatocellular carcinoma incidence. This figure is an enhanced version of Figure 2 in ref. 1 showing the adjusted ERS (aERS=ERS + 18.49) for human cancers. ‘Hepatocellular Average USA’ and ‘Hepatocellular Average TW’ denotes the entire population of HCC patients, including both HCV and non HCV cases, in the USA and in Taiwan, respectively. Incidence was taken from the SEER database. ‘Hepatocellular HBV’ denotes HCC patients who are also HBV carriers. HCC lifetime risk for HBsAg-positive patients was taken from ref. 13.

Tomasetti and Vogelstein did, however, consider risk-group specific incidences for a few cancers. For example, they calculated the excess risk score (ERS) for hepatocellular cancer (HCC) for the USA subpopulation infected by the hepatitis C virus (HCV) and the non HCV-infected subpopulation. The risk was 5.36 for HCV and -6.08 in non HCV cancers, which corresponded to the D-tumor (deterministic) and R-tumors (replicative) classes, respectively. This seems to support the validity of the ERS. But what would have been the classification of HCC if, as for most other cancers in the study, only the USA average incidence would have been taken into account? The ERS would be -5.65, well within the range of R-tumors, leading to the conclusion that HCC is a less preventable cancer ( Figure 1). This would be a dangerous distraction from the fact that 10 to 33% of them, depending on world regions, are caused by HCV infections that are both preventable and treatable when responsible health policies are implemented. Furthermore, is the non-HCV HCC not preventable, as its classification suggests? Fifty nine percent of HCC cases in the developing world are associated with hepatitis virus B (HBV) infection ^7^, which greatly increases the probability of developing the disease ( Figure 1). Universal HBV vaccination has resulted in a 65–75% reduction of HCC incidence in 6–14 years children from Taiwan ^8^. Other overlooked preventable risk factors for HCC include obesity, alcoholic cirrhosis, exposure to aflatoxin B and schistosomiasis. We focused on HCC due to space constrains, but similar arguments could be made for most cancers analyzed in ref. 1.

We also included in Figure 1 the ERS for the overall Taiwanese population. It is between the D- and R-tumors and higher than for the USA population. This is consistent with the fact that HCC is more preventable in Taiwan where HCV and HBV are more prevalent and supports, it seems, the potential usefulness of the ERS. Importantly, however, the ERS for all HCC rests on the same lscd estimate, thus the incidence data alone would produce the same ranking of the HCC groups [ERS=log _10_(lscd)×log _10_(incidence)]. On a more fundamental level, the ERS does not provide an absolute quantification of determinism because we do not know the baseline ERS for cancers occurring in the *proven* absence of any risk factor. Is this baseline universal or is it organ-specific? If the latter is correct, then the ERS will not be comparable among organs and will not be more informative than incidence data alone, as we have noted for HCC. If not, the ERS scale will be universal and the lscd will add information useful for the comparison of cancer determinism between organs. The modalities of DNA repair varies across the stem cell compartments of different organs ^9^, suggesting an organ-specific baseline.

To our knowledge, a substantial variation of the lscd in the general population cannot be excluded. Hence the stratification problem encountered for incidence data may also arise because of lscd variation. The authors wrote that factors “such as those that affect height and weight” could play a role. These factors are corroborated by epidemiology ^10, 11^. In addition, we consider highly plausible that tissue repair following chronic and possibly preventable damage may also significantly affect the lscd. Similarly, the relation between mutation rates and lscd is modulated by a range of factors, including DNA repair efficiency and activation of APOBEC DNA mutators ^12^. All of these were averaged out as were most known cancer risk factors.

In order to demonstrate the robustness of the correlation between the lscd and cancer lifetime risk, Tomasetti and Vogelstein varied randomly their lscd estimates over four orders of magnitude. We reproduced this calculation except that both lscd and incidences were varied randomly by two orders of magnitude (N=10,000). This calculation confirmed the robustness of the correlation (median *ρ*=0.54, 95% CI: 0.32–0.72; median p=0.002, 95% CI: 0.000005–0.08). We also collapsed to a single data point cancers sharing the same lscd estimate to address statistical independence concerns. Again, the correlation remained strong ( *ρ*=0.67, p=0.0009). The R script and the data to derive these results are given in supplementary material.

## Conclusion

The remarkable relation between cancer incidence and lscd uncovered by Tomasetti and Vogelstein is statistically robust. The ERS is typically high for known deterministic cancers. But we demonstrated that a cancer with a low ERS can include a sizable fraction of preventable diseases. This proves that their classification scheme, in its current form, is not suitable to gauge the likely effectiveness of prevention measures and to direct funding for research on cancer etiology. Many more risk factors for cancers will likely be discovered in the future. Hence, cancers ascribed to ‘bad luck’ today due to lack of proper risk stratification may someday become explainable and, hopefully, preventable.

R script and data to reproduce the analysis A description of each entry is provided in the readme file. Click here for additional data file. Copyright: © 2016 Tarabichi M and Detours V 2016 Data associated with the article are available under the terms of the Creative Commons Zero "No rights reserved" data waiver (CC0 1.0 Public domain dedication).

## Methods

Data were retrieved online from Table S1 of the supplementary material of ref 1.

Additional data were retrieved from ref 13. Incidence for HCC with HBV was computed as the sum of incidences in women and men, divided by 2 (HCC with HBV incidence=0.17685%). Lscd for all HCC was set to 2.709 10 ^11^, taken from ref 1.

Then data were analyzed in R v3.1.3 ^14^. Like in ref 1, both Spearman’s and Pearson’s correlation coefficients and *p*-values were computed. Here, we report values for Spearman’s correlations.

First, we reproduced Figure 2 of ref 1 after including data of HCC in Taiwan and with HBV. aERS were computed as described in ref 1, *i.e.* aERS=log _10_(lscd)×log _10_(incidence)+18.49.

Second, to assess the stability of the correlation between lscd and the incidence upon measurement errors, both lscd and incidence data were randomly multiplied or divided by 10, spanning two orders of magnitude. Incidences values were capped at 1. This was repeated 100 times for each variable, amounting to 100×100=10,000 pairs of randomly shifted lscd and incidences. From these 10,000 comparisons, distributions of correlation coefficients and *p*-values were obtained, from which confidence intervals were derived at percentiles 0.025 and 0.975.

Finally, we recomputed the correlation coefficient and *p*-value between lscd and incidence data after removing osteosarcomas and doublons, *i.e*. cancer originating from the same tissue type, thus having the same lscd. These doublons included *Colorectal adenocarcinoma with FAP*, *Colorecal adenocarcinoma with Lynch syndrome*, *Hepatocellular carcinoma with HCV*, *Head & neck squamous cell carcinoma with HPV-16*, *and Lung adenocarcinoma (smokers).*

## Data availability

The data referenced by this article are under copyright with the following copyright statement: Copyright: © 2016 Tarabichi M and Detours V

Data associated with the article are available under the terms of the Creative Commons Zero "No rights reserved" data waiver (CC0 1.0 Public domain dedication). 

F1000Research: Dataset 1. R script and data to reproduce the analysis, 10.5256/f1000research.9448.d133564 ^15^
