# Supplementary material for: A research note regarding "Variation in cancer risk among tissues can be explained by the number of stem cell divisions"
Source: F1000Res. 2016 Nov 10;5:2044. Originally published 2016 Aug 22. [Version 2] doi: 10.12688/f1000research.9448.2 (PMC5089134; doi:10.12688/f1000research.9448.2)
Supplement: Peer review history

# Peer review: Referee response for version 1

**DOI:** 10.5256/f1000research.10176.r16419

**Published:** 2016-10-25

**Authors:** Maddalena Fratelli, Marco Bolis

**Affiliations:** 1 Pharmacogenomics Unit, Department of Molecular Biochemistry and Pharmacology, IRCCS Mario Negri Institute for Pharmacological Research, Milano, Italy

This research note adds on the current discussion on the results of Tomasetti and Vogelstein, demonstrating that stochastic effects associated with stem cell division contribute in a substantial way to cancer incidence. As this conclusion has important and possibly harmful implications in the public health decisions, this deserves attention.

 On the one hand, this note confirms robustness of the T&V analysis of the correlation between lscd and cancer risk. On the other hand, it points out that cancers predicted to be replicative may include a proportion of preventable cases. This is demonstrated by the higher ERS index for hepatocellular carcinoma in Taiwan, where HCV and HBV prevalence, and therefore its preventability, is higher than in the USA.

 Minor points:

1. It would be interesting to show variation in ERS for other tumors such as, for example, esophageal and thyroid cancer. As discussed in Garattini & Tavani (2016), the incidence of these tumors show a great geographical variability. Even if it is not possible to associate these differences to preventable factors, this would add information on the global impact of geographical variation on ERS variability.
2. The discussion on lscd is also interesting. Perhaps the authors should make it explicit that more research is needed on the determination of the number of stem cell divisions and its dependence on factors such as different cell types in the same organ, damage, inflammation, individual height or weight and even ethnicity.
3. Finally, the authors should better explain why they deemed it important to demonstrate the robustness of T&V analysis, as they reproduced it with minor changes.

We have read this submission. We believe that we have an appropriate level of expertise to confirm that it is of an acceptable scientific standard.

# Peer review: Referee response for version 1

**DOI:** 10.5256/f1000research.10176.r16205

**Published:** 2016-09-23

**Authors:** Benjamin Roche, Cindy Gidoin

**Affiliations:** 1 Centre for Ecological and Evolutionary Research on Cancer, Montpellier, France; 2 Centre for Ecological and Evolutionary Research on Cancer, Université Montpellier, Montpellier Cedex, France

We think that this article could be an interesting addition to the current debate about the 'bad luck' theory. Briefly, this paper shows that the Adjusted Extra Risk Score (aERS) is dependent on the risk group that has been considered and that calculating this score at a country-scale level, without considering precisely all risk-groups, could give biased estimates. This suggests that prevention measures cannot be discarded (at least based only on this aERS), especially when a pathogen could be involved in cancer pathogenesis.

 We just have few minor remarks:

- In the results section, it is said that there is no relation between cancer and height or weight. Some references, especially with the Boyd-Orr cohort, exist; ^1^ ^2^
- We think it could be also worth mentioning that many cancers are due to infections, which therefore calls for a prudent interpretation before withdrawing prevention strategies against some cancers.

We have read this submission. We believe that we have an appropriate level of expertise to confirm that it is of an acceptable scientific standard.

## References

[1] AlbanesD: Height, early energy intake, and cancer. Evidence mounts for the relation of energy intake to adult malignancies. *BMJ*.1998;317(7169) :1331-2 981292410.1136/bmj.317.7169.1331PMC1114242

[2] AltmanAJSchwartzAD: Malignant diseases of infancy, childhood and adolescence. *Major Probl Clin Pediatr*.1978;18:1-515 651375
